# Gender discrepancy in research activities during radiology residency

**DOI:** 10.1186/s13244-019-0792-9

**Published:** 2019-12-21

**Authors:** Federica Vernuccio, Monika Arzanauskaite, Sevcan Turk, Estefania Terrazas Torres, Joanna Marie D. Choa, Ashlesha Satish Udare, Dina Haroun, Maria Mercedes Serra, Susan Shelmerdine, Bayarbaatar Bold, Jae Seok Bae, Eduardo Estades Romero, Valérie Vilgrain

**Affiliations:** 1 University of Palermo, Via del Vespro 129, 90127 Palermo, Italy; 2 Department ProMISE (Department of Health Promotion, Mother and Child Care, Internal Medicine and Medical Specialties), University Hospital of Palermo, Piazza delle Cliniche, 2, 90127 Palermo, Italy; 3 University Beaujon Hospital, University of Paris, Paris, France; 4 I.R.C.C.S. Centro Neurolesi Bonino Pulejo, Contrada Casazza, SS113, 98124 Messina, Italy; 5 Radiology and Imaging Department, Liverpool Heart and Chest Hospital, Liverpool, UK; 6 Cardiovascular Program ICCC, IR, HSCiSP, IIB-Sant Pau, Barcelona, Spain; 7 Radiology Department, Ege University Faculty of Medicine, 35100 Izmir, Turkey; 8 ICON Radiología e Imagen Diagnóstica, Hidalgo del Parral, Chihuahua Mexico; 9 Centro Médico ABC, 05330 Ciudad de México, CDMX, Mexico; 10 Institue of Radiology, St. Luke’s Medical Center-Global City, Taguig, Philippines; 11 MRI Department, Lilavati Hospital and Research Centre, Mumbai, India; 12 Radiology Department, Cairo University Hospitals, Cairo, Egypt; 13 Aswan Heart Center, Aswan, Aswan Governorate Egypt; 14 Departamento de Diagnos/co por Imagenes, Fleni. Montañeses 2325, C1428AQK, Ciudad de Buenos Aires, Argentina; 15 Department of Clinical Radiology, Great Ormond Street Hospital, London, WC1N 3JH UK; 16 UCL Great Ormond Street Institute for Child Health, London, WC1N 1EH UK; 17 Medical School, Etugen University, Ulaanbaatar, Mongolia; 18 Department of Radiology, Seoul National University Hospital, 101 Daehak-ro, Jongno-gu, Seoul, 03080 Republic of Korea; 19 Christiana Care Health System, Diagnostic Radiology, 4755 Ogletown-Stanton Road, Newark, DE 19718 USA; 20 Department of Radiology, University Hospitals Paris Nord Val de Seine, Beaujon, Clichy, Hauts-de-Seine, France; 21 CRI, UMR 1149, Inserm and Université Paris Diderot, Paris, France

**Keywords:** Gender, Residency, Mentorship, Training support

## Abstract

**Objective:**

To investigate the presence of gender disparity in academic involvement during radiology residency and to identify and characterize any gender differences in perceived barriers for conducting research.

**Methods:**

An international call for participation in an online survey was promoted via social media and through multiple international and national radiological societies. A 35-question survey invited radiology trainees worldwide to answer questions regarding exposure and barriers to academic radiology during their training. Gender differences in response proportions were analyzed using either Fisher’s exact or chi-squared tests.

**Results:**

Eight hundred fifty-eight participants (438 men, 420 women) from Europe (432), Asia (241), North and South America (144), Africa (37), and Oceania (4) completed the survey. Fewer women radiology residents were involved in research during residency (44.3%, 186/420 vs 59.4%, 260/438; *p* ≤ 0.0001) and had fewer published original articles (27.9%, 117/420 vs. 40.2%, 176/438; *p* = 0.001).

Women were more likely to declare gender as a barrier to research (24.3%, 102/420 vs. 6.8%, 30/438; *p* < 0.0001) and lacked mentorship/support from faculty (65%, 273/420 vs. 55.7%, 244/438; *p* = 0.0055). Men were more likely to declare a lack of time (60.3%, 264/438 vs. 50.7%, 213/420; *p* = 0.0049) and lack of personal interest (21%, 92/438 vs. 13.6%, 57/420, *p* = 0.0041) in conducting research.

**Conclusion:**

Fewer women were involved in academic activities during radiology residency, resulting in fewer original published studies compared to their men counterparts. This is indicative of an inherent gender imbalance. Lack of mentorship reported by women radiologists was a main barrier to research.

## Key points

- A significantly higher proportion of women radiology trainees perceive gender-based obstacles in research involvement during their radiology training program compared to male residents.
- Gender disparities in academic involvement during radiology residency have an impact on academic productivity: women trainees declare lower number of publications, namely original articles, compared to their men counterparts.
- Lack of adequate mentors and support from seniors are the most important perceived barriers to academic involvement for women radiology residents.

## Introduction

In the recent decades, advances of gender equity in medicine have resulted in a steady increase in the proportion of women physicians [1, 2]. However, radiology remains a male-dominated specialty in most countries with less than a third of women enrolled in radiology training or holding academic positions in the USA [3–5]. Furthermore, only 22–32% of women radiologists publish as first or last authors in radiology journals [6], only 13.7% of women are part of editorial boards in radiology journals [7], and, until today, no woman has been appointed as editor-in-chief of the main radiology journals in the last 16 years [8]. This is of concern because increased gender diversity helps to foster a more creative, productive, egalitarian, and innovative environment [8, 9].

An improved understanding of gender disparities in academic radiology would allow solutions to be formulated and implemented. Many potential influencing factors—i.e., maternity and household responsibilities, economic issues, time constraints, mentorship—have been investigated so far [10, 11]. Among these, discouragement from seniors, gender discrimination throughout a woman’s career [12–14], and inadequate academic mentorship seem to be critical [6, 15]. We hypothesize that gender discrepancy may be due to the relative unattractiveness of academic radiology to women residents and barriers to their advancement in academic radiology during specialty training and that this is an international trend. Our hypothesis is supported by the low proportion (~ 25%) of women involved in publications during radiology residency—which has not changed in the last decade [6]—as well as smaller start-up packages for women physician scientists [16]. Other authors have examined challenges related to involvement of residents in research [17–19], but, to our knowledge, no prior study has investigated involvement of women in academic opportunities during radiology residency.

The primary aim of this study was to investigate the presence of a gender disparity in academic involvement during radiology residency. A secondary objective was to identify and characterize any gender differences in perceived barriers for conducting research.

## Materials and methods

The study was carried out as an independent initiative by motivated participants of an “Introduction to Research for International Young Academics” program held at an international radiology conference. Ethical approval was not required for this study which involved voluntary participation in an anonymized prospective online survey of medical healthcare professionals.

### Questionnaire development

In order to assess differences in academic involvement and barriers to conducting research, a 35-question online survey was created using “Google Forms” (Google LLC, California, USA) by the lead authors in consensus to cover main issues surrounding academic involvement and shared as a web link that allows respondents to use any Internet browser. The questionnaire included a total of 35 questions (i.e., 33 multiple choice tick-box format questions and 2 open-ended questions). In addition to demographics and information on the training institution, the questionnaire covered the features of core radiology residency including:

- General information, including basic demographics, country where radiology residency was performed, year of radiology residency, size of training institution (i.e., small [< 100 beds], medium-sized [100–499], or large [≥ 500 beds] academic hospital), level of academic activity of training institution (i.e., not active if less than 5 scientific publications are published per year, moderately active if 5 to 20 publications are published per year, or very active if at least 20 publications are published per year), and family background in research/teaching
- Involvement in academic activities, including publication of thesis as medical student, poster and oral presentations at national and international conferences, scientific articles (i.e., review article, original article, and case report or case series) and publications as first author during radiology specialty training, and personal attitude towards research
- Barriers and personal willingness to participate to academic activities during residency and to perform a research fellowship after residency.

Gender-related questions were asked towards the end of the questionnaire to minimize implicit bias. An online link to access and complete the survey was generated for widespread distribution.

### Questionnaire distribution

All questionnaires were distributed with an introduction explaining the purpose of the survey and the target participants as well as instructions for those completing it, with the emphasis that the responses should reflect participants’ core radiology training and with added assurance that all responses would be anonymous. After the introduction, all the participants were asked to give their consent to participate. Our target audience were radiology trainees across the globe, including radiology residents, radiologists in current fellowship programs, or junior radiologists within 2 years after residency completion. We included all trainees that fulfilled the inclusion criteria and did not exclude any participants based on age or country of origin or training. In total, 876 participants responded to the online survey. Only surveys where there was no consent to participate (*n* = 6) or lack of response to the question of gender (*n* = 12) were excluded.

In order to reach as many trainees as possible, the following activities were carried out:

1. All co-authors personally contacted local radiology trainees within their own departments, city, and country where possible.
2. The online survey weblink was promoted via personal and professional social media avenues by co-authors which included Twitter and Facebook pages.
3. A formal request to distribute the survey was sent by the lead authors to 26 national and international radiological societies requesting the online link to be distributed to junior members of the societies and for permission to host the link on their society website.
  Of these, 17/31 (54.8%) societies agreed to distribute the survey (European Society of Oncologic Imaging [ESOI], European Society of Thoracic Imaging [ESTI], European Society of Cardiovascular Radiology [ESCR], Egyptian Society of Cardiovascular Radiology [EgSCR], and European Trainee Forum for Interventional Radiology of the Cardiovascular and Interventional Radiological Society of Europe [CIRSE]). National societies included the Argentinian [Argentina Society of Radiology, SAR], Belgian [Belgian Society of Radiology, BSR], Egyptian [Egyptian Society of Radiology and Nuclear Medicine, ESRNM], Italian [Italian Society of Medical Radiology (SIRM)], Lithuanian [Lithuanian Association of Radiologists – LRA], Mexican [Mexican Society of Radiology and Imaging, SMRI], Swiss [Swiss Society of Radiology, SSR], Turkish [Turkish Society of Radiology, TSR], and Korean [Korean Society of Radiology, KSR] radiology societies, American Institute of Radiologic Pathology [AIRM], Delaware Society of Radiology [DRS], and Asian & Oceanic Society for Paediatric Radiology [AOSPR]. Four societies declined to participate, and ten did not respond to the request or provided ambiguous answer without distributing the survey.

### Statistical analysis

Statistical analysis was performed using Excel (Microsoft Corp., Redmond, WA, USA) and GraphPad (San Diego, CA, USA) software. Descriptive statistics were used to analyze questions. Fisher’s exact or chi-square tests were used for comparisons, as appropriate. A *p* < 0.05 indicated a statistically significant difference.

Firstly, we tested whether there was any difference in the involvement in research activities depending on trainee gender. Specifically, differences in research output—including publications in conferences and journals—and in the attitude towards research were investigated. Secondly, we analyzed self-reported barriers to research and to post-residency research fellowship according to gender. A *p* < 0.05 indicated a statistically significant difference.

## Results

### Study cohort

**Fig. 1:**
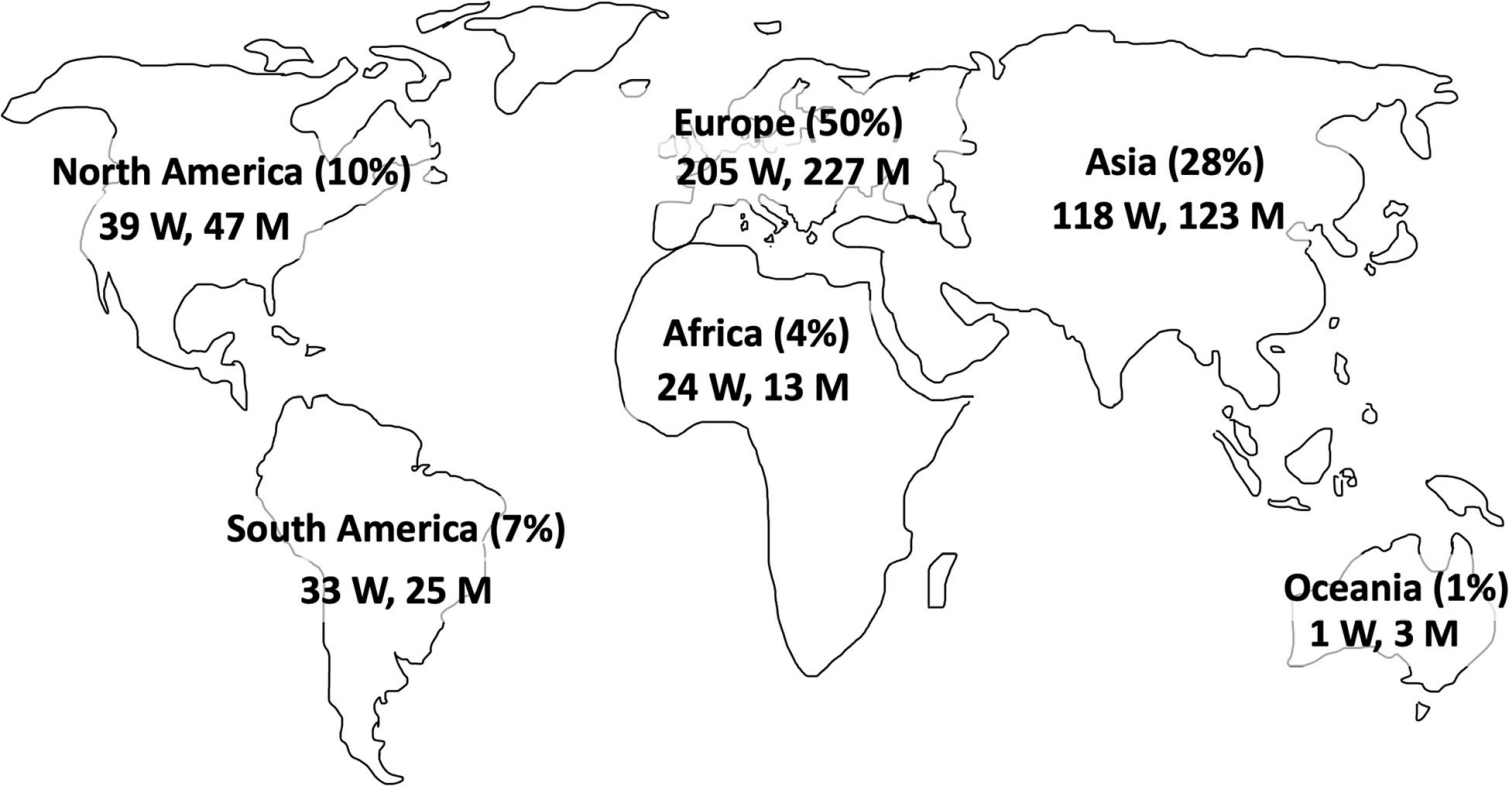
Participant distribution by continent. Percentages of overall participants per continent and raw data (numbers) of gender distribution are provided

The final study population included 858 participants (85.9% [737/858] aged 25–34 years old), including 438 (51%) men and 420 (49%) women. The per-continent distribution is summarized in Table 1, Fig. 1, and Additional file 1: Table S1. 58.0% (498/858) of the participants underwent their radiology residency program at a large academic hospital, and 54.4% (468/858) of the respondents described their institution as “moderately active” academically. 49.7% (426/858) of participants had no diversity and equality or bias training during radiology residency program, and 56.3% (483/858) and 65% (558/858) did not have flexible or part-time work opportunities, respectively.

**Table 1:** Study cohort: demographics and general information

	Women (*n* = 420)	Men (*n* = 438)	*p* value
Continents			
Europe	205 (49)	227 (52)	0.3772
Asia	118 (28)	123 (28)	0.9966
America	72 (17)	72 (16)	0.7826
North America	39 (9)	47 (11)	0.4814
South America	33 (8)	25 (6)	0.2103
Africa	24 (6)	13 (3)	0.0479*
Oceania	1 (< 0.01)	3 (1)	0.3371
Age range			
20–24	4 (1.0)	4 (0.9)	0.9525
25–29	158 (37.6)	173 (39.5)	0.5722
30–34	193 (46.0)	213 (48.6)	0.4325
35–39	48 (11.4)	33 (7.5)	0.0513
40–44	13 (3.1)	13 (3.0)	0.9135
45–49	4 (1.0)	2 (0.5)	0.384
Year of radiology residency			
First year	34 (8.1)	55 (12.6)	0.0322*
Second year	56 (13.3)	48 (11.0)	0.287
Third year	79 (18.8)	81 (18.5)	0.9054
Fourth year	72 (17.1)	103 (23.5)	0.0206*
Fifth year	31 (7.4)	33 (7.5)	0.932
I am in a subspecialty fellowship or PhD program	56 (13.3)	55 (12.6)	0.735
I completed my specialty and subspecialty training < 2 years ago	92 (21.9)	63 (14.4)	0.0042*
Institution			
Large Academic Hospital	239 (56.9)	259 (59.1)	0.5089
Medium Academic Hospital	144 (34.3)	147 (33.6)	0.8229
Small Academic Hospital	37 (8.8)	32 (7.3)	0.4184
Not active in research	84 (20.0)	93 (21.2)	0.6557
Moderately active in research	238 (56.7)	229 (52.3)	0.1977
Very active in research	98 (23.3)	116 (26.5)	0.2866

Categorical variables are provided as numbers and percentages. * indicates the statistically significant *p* values

### Gender disparities in academic involvement

Gender comparison showed significantly lower involvement of women in research compared to men (44.3% [186/420] vs 59.4% [260/438], respectively; *p* ≤ 0.0001), a lower number of papers presented at both national (19.3% [81/420] vs. 25.1% [110/438], respectively; *p* = 0.0403) and international conferences (14.3% (60/420) vs. 19.4 [85/438], respectively; *p* = 0.0455), and fewer published original articles, regardless of author’s order (27.9% [117/420] vs. 40.2% [176/438], respectively; *p* = 0.001) (Table 2). Overall, a significantly higher proportion of women reported a lower number of publications during residency compared to men (50.2% [211/420] vs. 42.5% [186/438], respectively; *p* = 0.0225).

**Table 2:** Research output and attitude towards research involvement during radiology training

	Women (*n* = 420)	Men (*n* = 438)	*p* value
Published thesis as medical student	99 (23.6)	120 (27.4)	0.2106
Doing research at the time of survey	186 (44.3)	260 (59.4)	< 0.0001*
Research output			
Presented at a poster at a conference	349 (83.1)	373 (85.2)	0.4081
Presented a poster at a national conference	203 (48.3)	216 (49.3)	0.7738
Presented a poster at an international conference	146 (34.8)	157 (35.8)	0.7402
Presented a paper at a conference	141 (33.6)	195 (44.5)	0.001*
Presented a paper at a national conference	81 (19.3)	110 (25%)	0.0403*
Presented a paper at an international conference	60 (14.3)	85 (19.4)	0.0455*
Published a scientific article in a journal	142 (33.8)	187 (42.7)	0.0075*
Published an original article	117 (27.9)	176 (40.2)	0.0001*
Published a review article	51 (12.1)	74 (16.9)	0.0487*
Published case report or case series	122 (29.0)	135 (30.8)	0.5708
Published an article as first author	133 (31.7)	154 (35.2)	0.2786
Published thesis as medical student	99 (23.6)	120 (27.4)	0.1991
No publications during residency	211 (50.2)	186 (42.5)	0.0225*
Family background in research/teaching	127 (30.2)	151 (34.5)	0.1852
Attitude towards research			
Improves clinical competency	251 (59.8)	269 (61.4)	0.6204
May compromise clinical competency	67 (16.0)	77 (17.6)	0.5239
Should be mandatory in training program	157 (37.4)	161 (36.8)	0.8503
It is important only for academic career	72 (17.1)	121 (27.6)	0.0002*
Willingness to perform research fellowship abroad	326 (77.6)	336 (76.7)	0.7518

Categorical variables are provided as numbers and percentages. * indicates the statistically significant *p* values

Although most trainees believe involvement in research during residency improves clinical competency (60.6% [520/858]), a significantly higher proportion of men compared to women believes that involvement in research is important only to pursue an academic career (27.6% [121/438] vs. 17.1% [72/420], respectively; *p* = 0.0002) (Table 2)

### Barriers to research

**Fig. 2:**
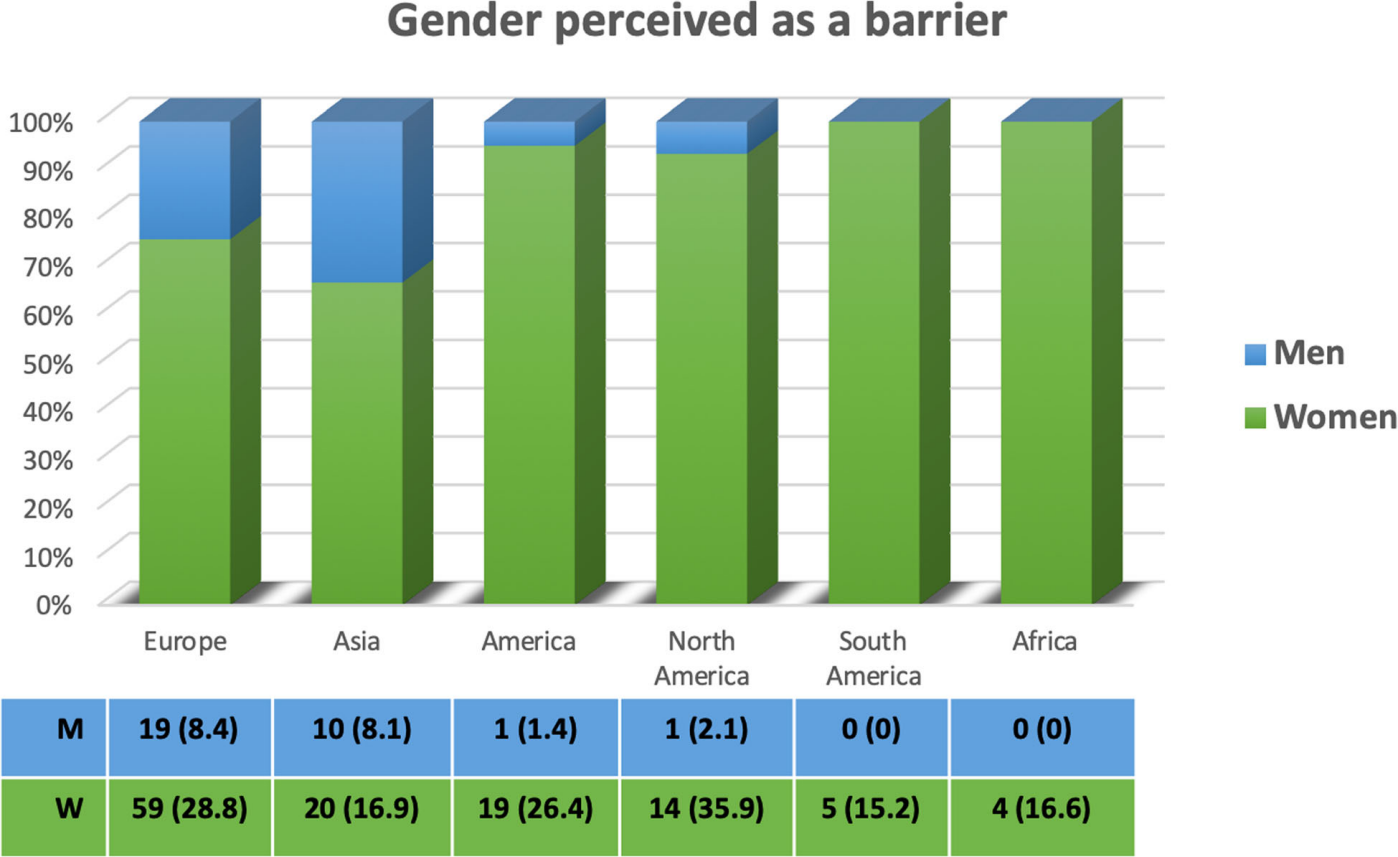
Gender perceived as barrier by continent. Raw data (numbers and percentages) are provided. Percentages in the columns are calculated relatively to the number of participants who declared gender as a barrier to research per continent. Percentages in the table are calculated relatively to the whole number of men and women who participated to the survey per continent

Gender comparison of barriers affecting involvement in research activities during residency demonstrated that a significantly higher proportion of women compared to men perceived gender as a barrier to research (24.3% [102/420] vs. 6.8% [30/438], respectively; *p* < 0.0001) (Table 3). Of note, the perception of gender as a barrier was more frequently reported by women than men regardless of their continent of origin although it was more pronounced in North America (14 of 39 women [35.9%]) and Europe (59 of 205 [28.8%]) than in Asia (20 of 118 [16.9%]), Africa ([4 of 24 [16.6%]), or South America (5/33 [15.2%]) (Fig. 2 and Additional file 1: Table S2).

**Table 3:** Barriers to research involvement during residency by gender

	Women (*n* = 420)	Men (*n* = 438)	*p* value
Barriers to research			
Lack of mentorship or support from faculty	273 (65.0)	244 (55.7)	0.0055*
Lack of time	213 (50.7)	264 (60.3)	0.0049*
Lack of research experience	157 (37.4)	148 (33.8)	0.2723
Lack of skills for statistical analysis	143 (34.0)	118 (26.9)	0.0238*
Lack of research ideas	113 (26.9)	114 (26.0)	0.771
Lack of funding	103 (24.5)	134 (30.6)	0.047*
Lack of reward	78 (18.6)	142 (32.4)	< 0.0001*
Frustration about complexity and slow progress	77 (18.3)	110 (25.1)	0.0162*
Lack of personal interest	57 (13.6)	92 (21.0)	0.0041*
Lack of opportunity to present research work	34 (8.1)	31 (7.1)	0.5736
Lack of access to libraries for research literature	22 (5.2)	28 (6.4)	0.4707
Barriers to perform research fellowship abroad			
Lack of funding	233 (55.5)	237 (54.1)	0.7316
Family circumstances/commitments	227 (54.0)	229 (52.3)	0.6321
It would result in reduction of my overall income	95 (22.6)	136 (31.1)	0.0056*
Lack of personal interest	93 (22.1)	116 (26.5)	0.1523
I do not see future possibilities after doing research	72 (17.1)	87 (19.9)	0.3339
I do not like living abroad	46 (11.0)	41 (9.4)	0.4976
I already did my research training as part of my core curriculum and it's sufficient	30 (7.1)	31 (7.1)	1.0000
Do you consider your gender as a challenge in research /teaching opportunities?			
Yes	102 (24.3)	30 (6.8)	< 0.0001*
No	318 (75.7)	408 (93.2)	< 0.0001*

Categorical variables are provided as numbers and percentages. * indicates the statistically significant *p* values

The top three barriers to research (lack of mentorship/support from faculty, lack of time, and lack of research experience) were the same factors reported by men and in women, although their order of importance differed. While women highlighted lack of mentorship/support from faculty (65% [273/420] vs. 55.7% [244/438], respectively; *p* = 0.0055), men were more concerned by the lack of time (men vs. women: 60.3% [264/438] vs. 50.7% [213/420], *p* = 0.0049). Among the other statistically significant barriers, lack of skills in statistical analysis and lack of personal interest were observed more commonly in women, while men considered lack of funding and lack of reward as barriers to research involvement more frequently than women (Table 3).

In the overall study cohort, the top three reasons preventing radiology trainees from undertaking a research fellowship abroad were lack of funding (54.8% [470/858]), family circumstances/commitments (53.1% [456/858]), and reduction of overall income (26.9% [231/858]). Reduction of overall income was perceived as a barrier more frequently by men compared to women (31.1% [136/438] vs. 22.6% [95/420], respectively; *p* = 0.0056).

## Discussion

This is the first study demonstrating self-reported gender disparity in academic involvement during radiology residency at an international level. Our study demonstrates that a significantly higher proportion of women radiology trainees perceive gender-based obstacles in research involvement during their radiology training program compared to male residents (24% vs. 7%, respectively). This was statistically significant for oral papers presented at radiological conferences as well as for publications of original and review articles. This phenomenon might be an explanation for the low number of women radiologists holding senior academic positions and who are involved in academic activities after residency. Prior studies have found similar results in other residency programs, including urology, where women urology residents produced fewer total publications (average 3.0 vs 4.8, *p* = 0.01) and fewer as first author (average 1.8 vs 2.5, *p* = 0.03) than men [20], and neurosurgery, where women also had statistically significantly lower research productivity assessed by several metrics (i.e., median publication count 4 [0–68] vs. 5 [0–198], *p* = 005; median *h*-index: 2 [0–16] vs. 2 [0–33], *p* = 0.022) [21].

It is well known that early participation in research encourages women to consider a future in academic medicine [11]. Specifically, participation in formal research training during residency is associated with decisions to pursue academic medicine and increases the likelihood of full-time faculty appointments for both genders [11]. Therefore, our results showing lower number of scientific publications in journals of women radiology trainees compared to their male counterparts support the findings of previous studies on gender differences in academic radiology after residency, including low number of women radiologists publishing as first or last authors and inadequate involvement of women in editorial boards of radiology journals [6, 7].

According to the results of our study, women and men have a similar attitude towards research and similar willingness to perform research fellowship abroad, which indicates that the differences in academic involvement are more related to the environment than individuals. The discrepancy in academic practice of radiology might not be resolved by passive intervention and, therefore, identification of gender-related barriers radiology trainees face in academic involvement is of utmost importance. In accordance with previous robust literature on women in academic medicine [11, 15], our study demonstrates that lack of adequate mentors and support from seniors are the most important perceived barriers to academic involvement for women radiology residents. Consequently, formal mentoring of women is an important potential resource to increase the proportion of women residents pursuing academic careers and positions of leadership [8, 22–25]. A recent experience at Indiana University, where a women specific mentoring program for radiology residents, fellows, and practicing radiologists was created, achieved promising results with increased networking and research involvement of radiology trainees [26]. In addition, in 2016 the #RADWomen initiative—otherwise known using the hashtag #RadXX—has been launched on Twitter. This movement focuses on fostering networking and mentorship opportunities for women involved in radiology, informatics, and radiology systems IT management, and it has demonstrated significant potential for conversation, debate, and collaborative learning, while expanding the reach of ideas and networks [27].

As shown by the results of this study, the radiological community should work to further promote a positive cultural shift towards research and gender balance during radiology training at different levels. There is no single straightforward solution but we provide possible suggestions. At an institutional level, a good start would be to introduce and encourage research activities during medical school, to include research work as a part of radiology residency program with protected time for research and to clearly and publicly state values of equality and diversity in research. At a department level, chiefs should promote gender balance by avoiding all-men radiological panels (“manels”), initiate an audit or a quality improvement project on gender equity, and, if needed, plan actions to promote gender balance including equal salary, rewards, and mentoring opportunities. In regard of promoting research involvement, chiefs should offer a half day of research time for those who are academically interested, include courses on how to perform research (e.g., integrity in conducting research, data collection, literature research, basic medical statistics, manuscript writing) in the formal teaching program, provide an appropriate reward for trainees who are keen on doing research, and ensure that academic achievements of the department are regularly highlighted in newsletters, emails, or departmental social media accounts. Supervisor and mentors are strongly needed in order to guide and motivate trainees and should focus on the following attitude: ensure that protected time for research is provided and respected, encourage trainees to participate in research by suggesting tasks within a project already happening, start and/or supervise a local journal club or monthly research meetings, provide positive constructive feedbacks, and make trainees aware of any research awards/prizes/grants that a trainee is eligible for. In addition, supervisors and mentors of the department should respect a gender balance in the research team and productivity. Last, but not least, trainees should show that they are keen to participate to research, enquire their colleagues and supervisors whether there is any project they could help, and ask to present/discuss a recently published paper at a local journal club. Finally, if trainees are selected for a team or project, and there is inadequate gender balance, they should speak up and report this to the supervisor, mentor, and/or chief.

Some limitations pertain to this study. Firstly, the questionnaire was not linked to local or institutional training programs and did not assess perceived workload in clinical and academic parts of training. However, these analyses were beyond the scope of this study, which was focused on self-reported gender disparities during radiology residency and not on the quality of institutional training programs. Secondly, given the online distribution of the questionnaire, we cannot assess how many trainees read the advertisement and chose to not participate and how many residents did not receive the call participation. We acknowledge that some national and international radiology societies refused to send the call for participation or ignored our request, which may have limited distribution to certain subspecialty and country groups. In addition, the use of Google LLC, California, USA, is banned in some countries and is not accessible via some hospital institutions through their online network. Therefore, our cohort may not reflect all international radiology trainees as a whole and our study might have had a broader implication if these limitations were not present. Nevertheless, social media—i.e., Twitter and Facebook—has become a tremendous vehicle for communication among radiology trainees [28], and this helped to balance some of these issues. Thirdly, information on the relationship, parental, marital, or child-bearing responsibilities of each radiology trainee was not assessed, which prevented evaluation of these confounder factors of gender differences. Surprisingly, lack of time was considered a barrier to research more frequently by men compared to women, and there was not a significant difference between the two genders in declaring family circumstances as a barrier to performing a research fellowship, suggesting that these family responsibilities do not prevent women from dedicating time to research. Nonetheless, these factors may be particularly relevant during the final years of residency or soon after residency and deserve careful further analysis.

In conclusion, this is the first study demonstrating that women radiology residents perceive gender disparities in research involvement at an international level. Women radiology residents declare a greater self interest in research compared to men, but are less involved in research activities, citing lack of mentorship and support from seniors as a key barrier resulting in fewer published original and review articles. Active intervention is needed, which should start with implementation of allocated time for research and mentorship programs for women trainees.

## Additional file

**Additional file 1: Table S1.** Response rate and gender distribution of participants by country. **Table S2.** Number of participants declaring their gender as a barrier in research/teaching opportunities in countries with at least 20 participants.

## Data Availability

The datasets used and/or analyses during the current study are available from the corresponding author on reasonable request.
