# Supplementary material for: Gender discrepancy in research activities during radiology residency
Source: Insights Imaging. 2019 Dec 21;10:125. doi: 10.1186/s13244-019-0792-9 (PMC6925606; doi:10.1186/s13244-019-0792-9)
Supplement: Supplementary file 1 — Additional file 1: Table S1. Response rate and gender distribution of participants by country. Table S2. Number of participants declaring their gender as a barrier in research/teaching opportunities in countries with at least 20 participants. [file 13244_2019_792_MOESM1_ESM.docx]

**Additional file 1**

**Additional file 1: Table S1.** Response rate and gender distribution of participants by country.

| **Country** | **N of participants** | **N of women** |
| --- | --- | --- |
| Algeria | **3** | **2** |
| Argentina | 57 | 33 |
| Australia | 3 | 1 |
| Austria | 2 | 2 |
| Bahrain | 1 | 1 |
| Bangladesh | 1 | 1 |
| Belgium | 19 | 11 |
| Bulgaria | 1 | 1 |
| Canada | 3 | 1 |
| Croatia | 2 | 1 |
| Czech Republic | 1 | 0 |
| Cuba | 1 | 0 |
| Denmark | 17 | 11 |
| Egypt | 26 | 19 |
| Ethiopia | 1 | 0 |
| Finland | 1 | 1 |
| France | 10 | 2 |
| Germany | 2 | 0 |
| Greece | 11 | 4 |
| Holland | 1 | 0 |
| Hungary | 2 | 1 |
| India | 29 | 13 |
| Indonesia | 4 | 0 |
| Iraq | 1 | 1 |
| Italy | 239 | 119 |
| Jordan | 1 | 1 |
| Kenya | 1 | 1 |
| Korea | 21 | 7 |
| Latvia | 5 | 3 |
| Lebanon | 3 | 0 |
| Libya | 1 | 0 |
| Lithuania | 52 | 21 |
| Malaysia | 1 | 1 |
| Mexico | 27 | 13 |
| Mongolia | 48 | 34 |
| Morocco | 1 | 0 |
| Netherlands | 9 | 6 |
| New Zealand | 1 | 0 |
| Nigeria | 1 | 1 |
| Norway | 2 | 1 |
| Pakistan | 2 | 2 |
| Paraguay | 1 | 0 |
| Philippines | 57 | 30 |
| Poland | 2 | 1 |
| Portugal | 1 | 0 |
| Romania | 3 | 3 |
| Russia | 2 | 2 |
| Saudi Arabia | 5 | 3 |
| Serbia | 2 | 2 |
| Singapore | 4 | 0 |
| South Africa | 1 | 0 |
| Spain | 10 | 3 |
| Sweden | 3 | 2 |
| Switzerland | 7 | 2 |
| Taiwan | 1 | 0 |
| Thailand | 4 | 1 |
| Tunisia | 2 | 1 |
| Turkey | 55 | 23 |
| UK | 28 | 7 |
| United States | 55 | 25 |
| Yemen | 1 | 0 |

**Additional file 1: Table S2.** Number of participants declaring their gender as a barrier in research / teaching opportunities in countries with at least 20 participants.

|  | Women  N (%) | Men  N (%) |
| --- | --- | --- |
| Argentina (33 W, 24 M) | 5 (15.2) | 0 (0) |
| Egypt (19 W, 7 M) | 2 (10.5) | 0 (0) |
| India (13 W, 16 M) | 2 (15.4) | 3 (18.7) |
| Italy (119 W, 120 M) | 44 (37.0) | 12 (10.0) |
| Korea (7 W, 14 M) | 1 (14.3) | 1 (7.1) |
| Lithuania (21 W, 31 M) | 4 (19.0) | 2 (6.5) |
| Mexico (13 W, 14) | 2 (15.4) | 0 (0) |
| Mongolia (34 W, 14 M) | 8 (23.5) | 1 (7.1) |
| Philippines (30 W, 27 M) | 0 (0) | 0 (0) |
| Turkey (23 W, 29 W) | 9 (39.1) | 3 (10.3) |
| UK (7 W, 21 W) | 0 (0) | 1 (4.8) |
| United States (25 W, 30) | 12 (48) | 0 (0) |
